# Tracing GFP-labeled WJMSCs in vivo using a chronic salpingitis model: an animal experiment

**DOI:** 10.1186/s13287-017-0714-z

**Published:** 2017-12-01

**Authors:** Zhe Li, Zhao Zhang, Wai-kit Ming, Xin Chen, Xiao-min Xiao

**Affiliations:** 1 The Department of Obstetrics and Gynecology, 1st Affiliated Hospital of Jinan University, Guangzhou, 510000 China; 2 The Department of Reproduction, Southern Medical University Affiliate Dongguan People’s Hospital, Dongguan, China; 3 Harvard Medical School, Harvard University, Boston, MA USA

**Keywords:** Green fluorescent protein, Wharton’s jelly-derived mesenchymal stem cells, Tracing, Oviducts, Transformation

## Abstract

**Background:**

The present study was conducted to evaluate the distribution of Wharton’s jelly-derived mesenchymal stem cells (WJMSCs) and their repairing function on the oviduct.

**Methods:**

WJMSCs were transfected with the LV3-GFP-PURO lentivirus. Female New Zealand rabbits (*n* = 24) were divided randomly into control A and B groups and experimental C and D groups to establish inflammation models. Sterile saline solution or WJMSCs were injected into rabbits via ear veins and/or genital tract perfusion once weekly for 3 weeks. All rabbits were humanely sacrificed 1 week after the last perfusion to collect the oviduct, uterus, liver, and bladder for examination. Green fluorescent protein (GFP) and cytokeratin 7 (CK7) were imaged using a Leica Qwin Plus V3 fluorescence confocal microscope and analyzed as mean optical densities in an Image-Pro Plus analysis system.

**Results:**

We found that lentivirus expressing the GFP gene produced an efficient transfection. The mean optical density values of GFP and CK7 in the oviducts were higher in the experimental D group than those in the control A and experimental C groups. No GFP fluorescence deposits occurred in the bladder of the control A group or experimental C group. Colocalization of CK7 and WJMSCs was observed in the oviducts in all groups.

**Conclusions:**

WJMSCs exhibited homing characteristics and migrated to the injured oviduct to promote epithelial cell growth. Additionally, local treatment resulted in higher efficiency.

## Background

Tubal infertility has long been considered the major cause of female infertility. Salpingitis and/or pelvic inflammation caused by infection is one of the most important factors of tubal infertility. Severe salpingitis may damage the fallopian tube mucosa, and pelvic inflammation damages the structure of the oviduct, which may result in fimbria adhesion, distal tube obstruction, and hydrosalpinx [1]. However, current treatment methods cannot fix the tube injury. Therefore, new methods to repair the reproduction function of the oviduct are required to allow gametes to combine and grow in vivo under natural circumstances.

Mesenchymal stem cells (MSCs) [2] are used in cell therapy and regenerative medicine because these cells are easily isolated and acquired, exhibit rapid expansion in culture, can be used in autologous transplantation, and exhibit significant paracrine effects [3]. Wharton’s jelly-derived mesenchymal stem cells (WJMSCs) possess distinct advantages, such as accessibility, painless donation procedures, and high separation rates [4]. Moreover, WJMSCs do not express main histocompatibility complex (MHC) II [5] and exhibit low immunogenicity and little or no expression of MHC I [6]. WJMSCs exhibit a higher proliferation capacity and lower expression of CD106, HLA-ABC, and HLA-DR than MSCs from bone [7, 8]. Previous studies have shown that WJMSCs could survive in vivo for a comparatively long time after engraftment [9–11], due to their ability to modulate the immunological response contributing to WJMSC viability [12, 13].

Therefore, our study established a chronic salpingitis model in New Zealand rabbits and transplanted WJMSCs expressing green fluorescent protein (GFP) using different methods to evaluate the distribution of these stem cells. Our hope is that the findings from this study will further inform and guide the future clinical use of WJMSCs to treat tubal infertility in women.

## Methods

### Isolation, culture, and identification of WJMSCs

Human umbilical cord tissue was obtained from healthy and full-term infants who were born via social-factor caesarean section. HbsAg, anti-HIV, CMV-IgM, syphilis, mycoplasma, and chlamydia tests were negative. The umbilical cord tissue was washed with D-Hanks BSS. Umbilical veins, the umbilical artery, and the outer membrane of the umbilical tissue were dislodged. Wharton’s jelly was removed and cut into 1 mm × 1 mm × 1 mm tissue blocks. These tissue blocks were resuspended in 0.075% type I collagenase and incubated at 37 °C for 10–14 h with magnetic stirrers. The digested mixture was washed and diluted in D-Hanks BSS, and the suspensions were centrifuged at 1500 rpm for 5 min at room temperature to obtain a cell pellet. The pellet was washed and centrifuged three times in D-Hanks BSS. The pellet was washed and resuspended in growth medium containing DMEM and 10% FBS and was cultured in 5% CO_2_ in a 37 °C incubator. The growth medium was renewed every 3 days. Cells that adhered to the wall were fusiform fibroblasts and were 80% fused in approximately 1 week. Flow cytometry was used to detect the presence of CD73, CD90, and CD105 and the absence of CD34 and CD45 to determine which cells were WJMSCs. The cell viability in our study was 95–98%, and cells were diluted with a sterile saline solution to 1 × 10^6^/ml on the day of study. All of these materials were manufactured and provided by the Cord Blood Bank of Guangdong Province of China on the day of use.

### Model-making bacterial strain

A lyophilized (ATCC25922) strain was inoculated in a fresh beef infusion broth and cultured in an incubator (37 °C) for 24 h. The mixture was centrifuged at 1000 rpm for 10 min at room temperature to obtain precipitates. The precipitates were washed three times in PBS and diluted with a sterile saline solution to a 3 × 10^8^/ml *Escherichia coli* suspension.

### Establishment of the animal model in pre-experiments

As our previous work demonstrated before, the trans-vaginal intrauterine administration of a 3×10^8^/ml Escherichia coli suspension was sufficient to establish a chronic salpingitis model in female New Zealand rabbits. Salpingitis became a chronic inflammatory condition in 15 days [14].

### Experimental animals and grouping

Twenty-four pathogen-free female New Zealand rabbits (4–5 months old, nonpregnant, weighing 2.5 kg ± 250 g) were provided by the Animal Experiment Center of Guangzhou University of Chinese Medicine (qualification no. CV20130015). All experimental animals were divided equally into two groups: a control group and an experimental group. All female rabbits were injected with 80 IU of HCG to synchronize the estrous cycles. Female rabbits in the experimental group were anesthetized using pentobarbital sodium. Disposable sterile newborn sputum suction tubes were inserted into the urogenital tract to instill an *E. coli* suspension into the uterine cavity to establish the chronic salpingitis model.

Rabbits in the control group (*n* = 12) were divided randomly into A and B groups. Fifteen days after HCG injections, rabbits in the control A group (*n* = 6) were injected with 0.5 ml of a 1 × 10^6^/ml GFP-labeled WJMSC suspension via the ear vein. Disposable sterile newborn sputum suction tubes were inserted into the urogenital tract of rabbits to instill 0.5 ml of a 1 × 10^6^/ml GFP-labeled WJMSC suspension. This procedure was executed once weekly for 3 weeks. Rabbits in the control B group (*n* = 6) received 1.0 ml of a 1 × 10^6^/ml GFP-marked WJMSC suspension via the urogenital tract. This procedure was also executed once weekly for 3 weeks [15–20]. All 12 rabbits were humanely sacrificed 1 week after the last WJMSC perfusion, and the oviduct, uterus, bladder, and liver were sampled for examination.

Experimental groups (*n* = 12) were perfused with an *E. coli* suspension and divided randomly into C and D groups. Fifteen days after intubation, rabbits in the experimental C group were injected with 0.5 ml of a 1 × 10^6^/ml suspension of GFP-marked WJMSCs, via the ear vein. Disposable sterile newborn sputum suction tubes were inserted into the urogenital tract of rabbits to perfuse 0.5 ml of a 1 × 10^6^/ml suspension of GFP-labeled WJMSCs. This procedure was executed once weekly for 3 weeks. Rabbits in the experimental D group received 1.0 ml of a 1 × 10^6^/ml suspension of GFP-labeled WJMSCs via the urogenital tract. This procedure was also executed once weekly for 3 weeks. All 12 rabbits were humanely sacrificed 1 week after the last WJMSC perfusion, and the oviduct, uterus, bladder, and liver were sampled for examination.

One female rabbit in the control group died on the 20th day of the experiment, and one female rabbit in the experimental group died on the 23rd day of the experiment. The other 22 rabbits exhibited normal appearance and vaginal secretions. No antibiotics or drugs were administered during the experimental period.

### Cell transfection and transfection efficiency

The LV3-GFP-PURO lentivirus (5 × 10^9^ TU/ml) was purchased from GenePharma (China). WJMSCs were inoculated into two plates with six wells each 1 day before transfection. Each well contained 2 × 10^5^ WJMSCs. When the cells reached 70–80% confluence, the complete medium (DMEM-high glucose + 10% FBS) was removed. WJMSCs were then transfected with LV3-GFP-PURO lentivirus (5 × 10^9^ TU/ml at 100, 200, and 400 MOI in the presence of 5 μg/ml polybrene and complete medium). GFP was measured using flow cytometry at 96 h to evaluate transfection efficiency.

### Assessment of cell proliferation ability

Assessment of WJMSC proliferation ability was performed using the MTT assay. WJMSCs were inoculated into four plates with 96 wells per plate. Each well contained a density of 2 × 10^5^ WJMSCs. The WJMSCs were divided into transfer and untransfected groups. The 96-well plates were placed in an incubator at 5% CO_2_ at a temperature of 37 °C overnight. WJMSCs in the transfer group were transfected with LV3-GFP-PURO lentivirus (5 × 10^9^ TU/ml) at 200 MOI based on the results of transfection efficiency, and WJMSCs in the untransfected group received an equivalent dose of PBS. Each group had five parallel wells. Cells in one of the four plates were incubated with the MTT solution for 4 h at 24, 48, 72, and 96 h after transfection. The medium was removed, and 150 μl of dimethylsulfoxide was added to each well. Absorbance was measured at 490 nm using a Model 680 microplate reader.

### GFP-labeled WJMSC suspensions

WJMSCs transfected with lentivirus at 200 MOI for 96 h were cultured in selection medium (DMEM-high glucose + 10% FBS + 2 μg/ml puromycin) to select positive cells. Untransfected cells were washed away 1 day after selection, and transfected cells were cultured in new selection media. The remaining cells were cultured in normal media 2 days after the second selection. Cells were cultured to 80–90% confluence, and the transfected WJMSCs were trypsinized and diluted with a sterile saline solution to 1 × 10^6^/ml for experimental use.

### Frozen sectioning and immunofluorescent staining

Fresh tissue cryosections (3–4 μm) were embedded in optimum cutting temperature compound gel and fixed for 10 min in 4 °C acetone.

Sections were then washed in PBS three times for 5 min. CK7 (1:100) primary antibodies were added to sections and incubated at 37 °C in an incubator for 1 h.

Afterwards, sections were washed three times in PBS. Fluorogenic secondary antibodies were added to sections and incubated at 37 °C for 1 h.

Sections were then washed again in PBS and mounted in Fluoromount-G. Stained sections were imaged using a Leica Qwin Plus V3 fluorescence confocal microscope and analyzed as mean optical densities (MODs) in an Image-Pro Plus analysis system.

### Statistical methods

All data are presented as the mean ± standard error. Assessments of cell proliferation ability were analyzed using independent-sample *t* tests, and *P* < 0.05 was considered statistically significant. Other outcomes were analyzed using one-way-ANOVA and the least-significant difference test (homogeneity of variance) or Tamhane’s T2 test (heterogeneity of variance). All data were analyzed using SPSS 13.0 statistical software (SPSS, Inc., Chicago, IL, USA).

## Results

### Assessment of transfection efficiency of LV3-GFP-Puro lentivirus in vitro

**Fig. 1:**
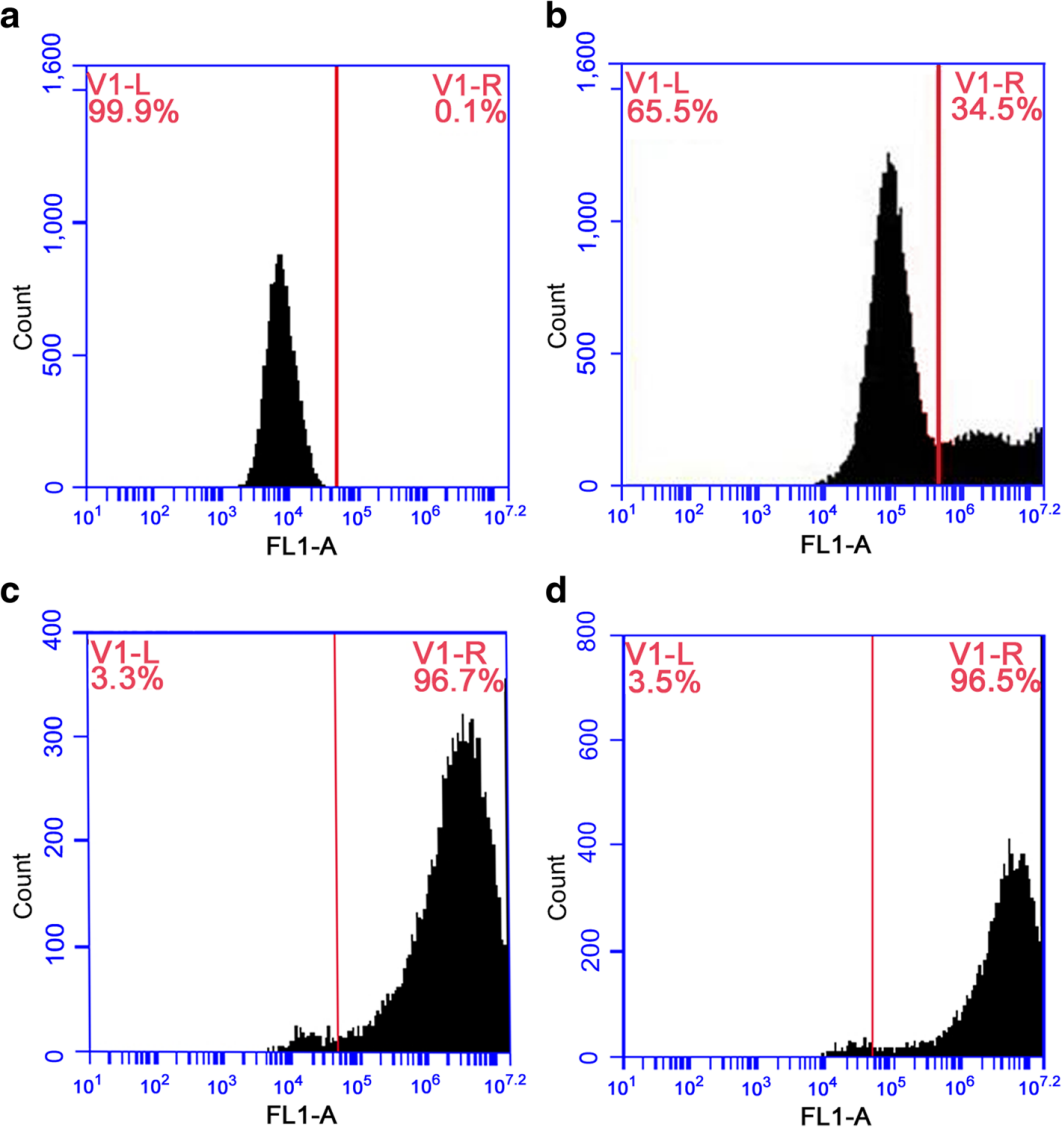
Assessment of transfection efficiency using flow cytometry. **a** Untransfected cells. **b** Transfection of cells at MOI = 100. **c** Transfection of cells at MOI = 200. **d** Transfection of cells at MOI = 400

Cells were transduced with LV3-GFP-PURO lentivirus (5 × 10^9^ TU/ml) at 100, 200, and 400 MOI for 96 h, and all WJMSCs expressed green fluorescence. Fluorescence intensity increased with increasing MOI values. Flow cytometry demonstrated that the transfection efficiency was 34.5% at 100 MOI, 96.7% at 200 MOI, and 96.5% at 400 MOI. Optimal transfection efficiency of LV3-GFP-Puro lentivirus occurred at 200 MOI, and 200 MOI was therefore used in subsequent experiments (Fig. 1).

### Assessment of cell proliferation ability in vitro

Assessment of WJMSC proliferation ability was performed using the MTT assay. The results demonstrated that the cell proliferation ability of WJMSCs in the transfected group and the untransfected group was not significantly different at any time (Table 1).

**Table 1:** Cell proliferation ability in the transfer group and the untransfected group

Group	24 h (OD)	48 h (OD)	72 h (OD)	96 h (OD)
Transfer	0.31 ± 0.03	0.42 ± 0.02	0.64 ± 0.06	1.01 ± 0.06
Untransfected	0.31 ± 0.02	0.40 ± 0.01	0.60 ± 0.02	0.94 ± 0.05
*P*	0.683	0.107	0.213	0.069

*OD* optical density

### Tracking of WJMSCs in vivo

#### MOD of GFP in rabbit oviducts

**Fig. 2:**
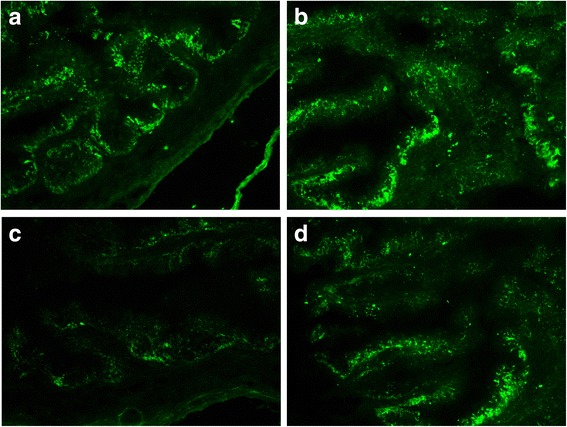
GFP fluorescence deposits in the epithelia of the oviduct. **a** Control A group. **b** Control B group. **c** Experimental C group. **d** Experimental D group

GFP fluorescence deposits occurred in the epithelia of the oviduct. There were no deposits in mesenchymal or muscular layers. One-way ANOVA and Tamhane’s T2 test (heterogeneity of variance, *P* < 0.05) were performed to examine the MOD values of GFP in the oviducts of different groups. The MOD values of GFP in the oviducts of the different groups were significantly different (*P* < 0.05). MOD values of GFP in the experimental D group were higher than in the control A group and experimental C group (*P* = 0.037, *P* = 0.047). No significant difference were found between the control A group and the experimental C group (*P* = 0.147) (Fig. 2, Table 2).

**Table 2:** Mean optical density of GFP in different rabbit groups

		MOD			
Group	*n*	Oviduct	Uterus	Bladder	Liver
Control A	5	56.95 ± 2.26	40.03 ± 22.78	0.00 ± 0.00	80.62 ± 13.49
Control B	5	62.45 ± 3.93	54.71 ± 0.85	49.34 ± 2.40	66.87 ± 21.05
Experimental C	6	59.29 ± 7.58	37.22 ± 28.85	0.00 ± 0.00	69.43 ± 3.50
Experimental D	6	82.94 ± 14.37	57.91 ± 9.53	51.86 ± 6.45	64.59 ± 1.45
*F*		10.549	1.633	368.295	1.843
*P*		0.000	0.217	0.000	0.176

*GFP* green fluorescent protein, *MOD* mean optical density

#### MOD of GFP in rabbit uteri

**Fig. 3:**
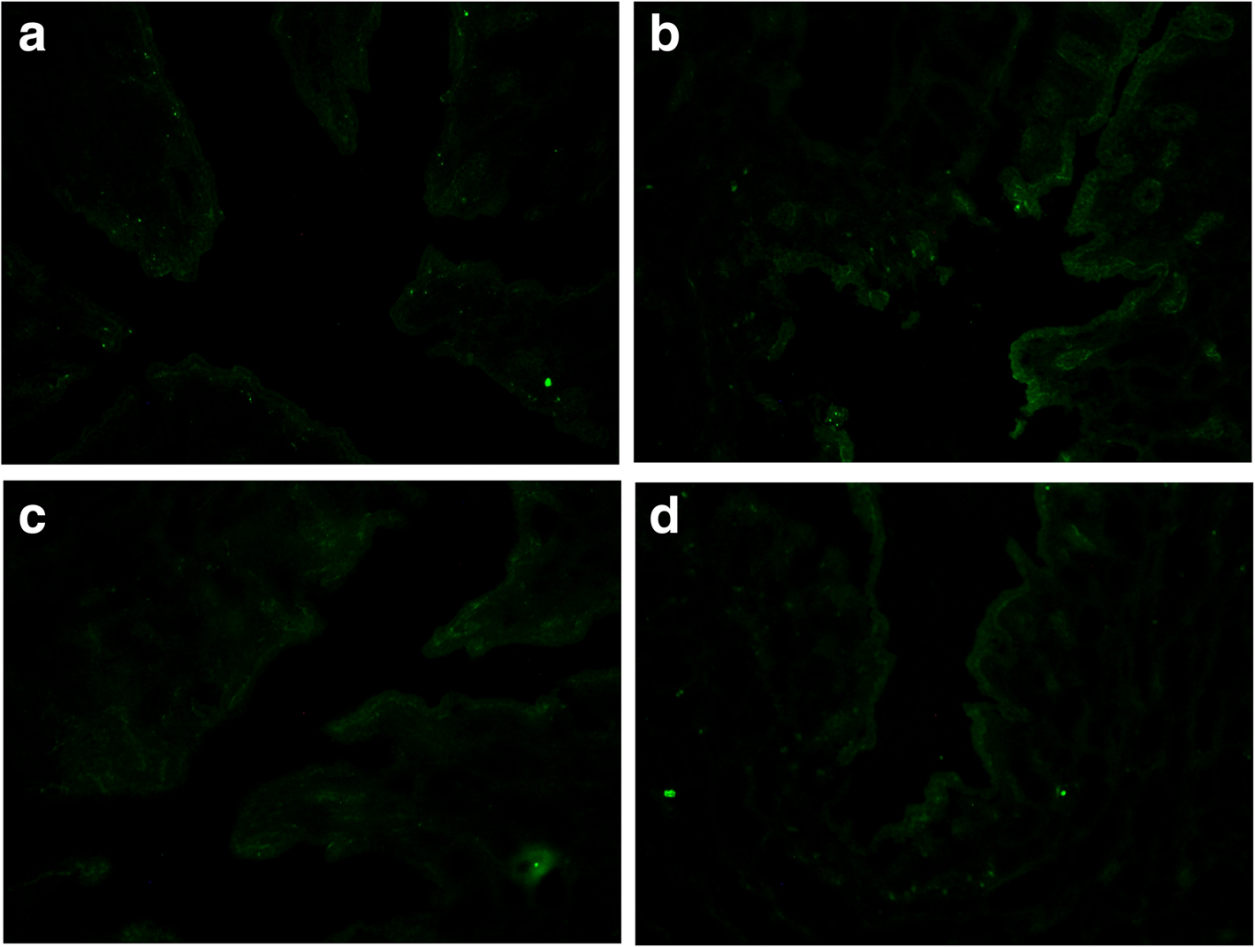
GFP fluorescence deposits in the endometrium. **a** Control A group. **b** Control B group. **c** Experimental C group. **d** Experimental D group

GFP fluorescence deposits occurred in epithelial cells in the endometrium. There were no deposits in the muscular layer. Two rabbits in the control A group had no fluorescence deposits in the endometrium. One-way ANOVA revealed that the MOD values of GFP in the uteri of different groups were not significantly different (*P* = 0.217) (Fig. 3, Table 2).

#### MOD of GFP in rabbit bladders

**Fig. 4:**
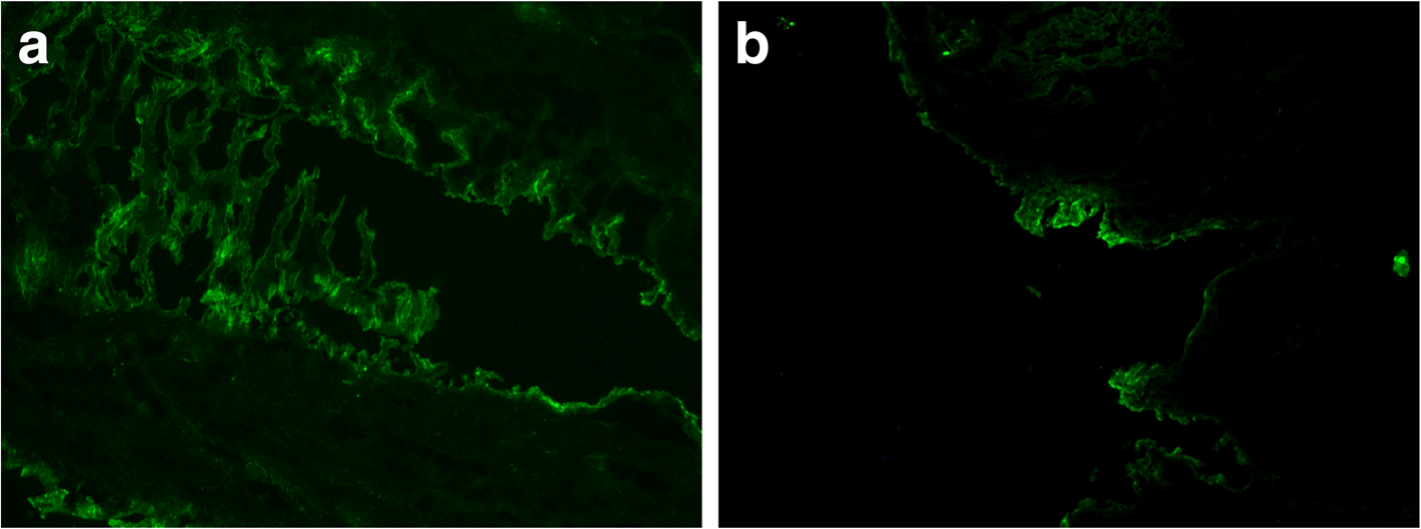
GFP fluorescence deposits in the epithelia of the bladder. **a** Control B group. **b** Experimental D group

GFP fluorescence deposits occurred in the epithelia of the bladder. No GFP fluorescence deposits occurred in the control A group or experimental C group. One-way ANOVA and Tamhane’s T2 test (heterogeneity of variance, *P* < 0.05) revealed that the MOD values of GFP in the bladders of different groups were significantly different (*P* < 0.05) (Fig. 4, Table 2).

#### MOD of GFP in rabbit livers

**Fig. 5:**
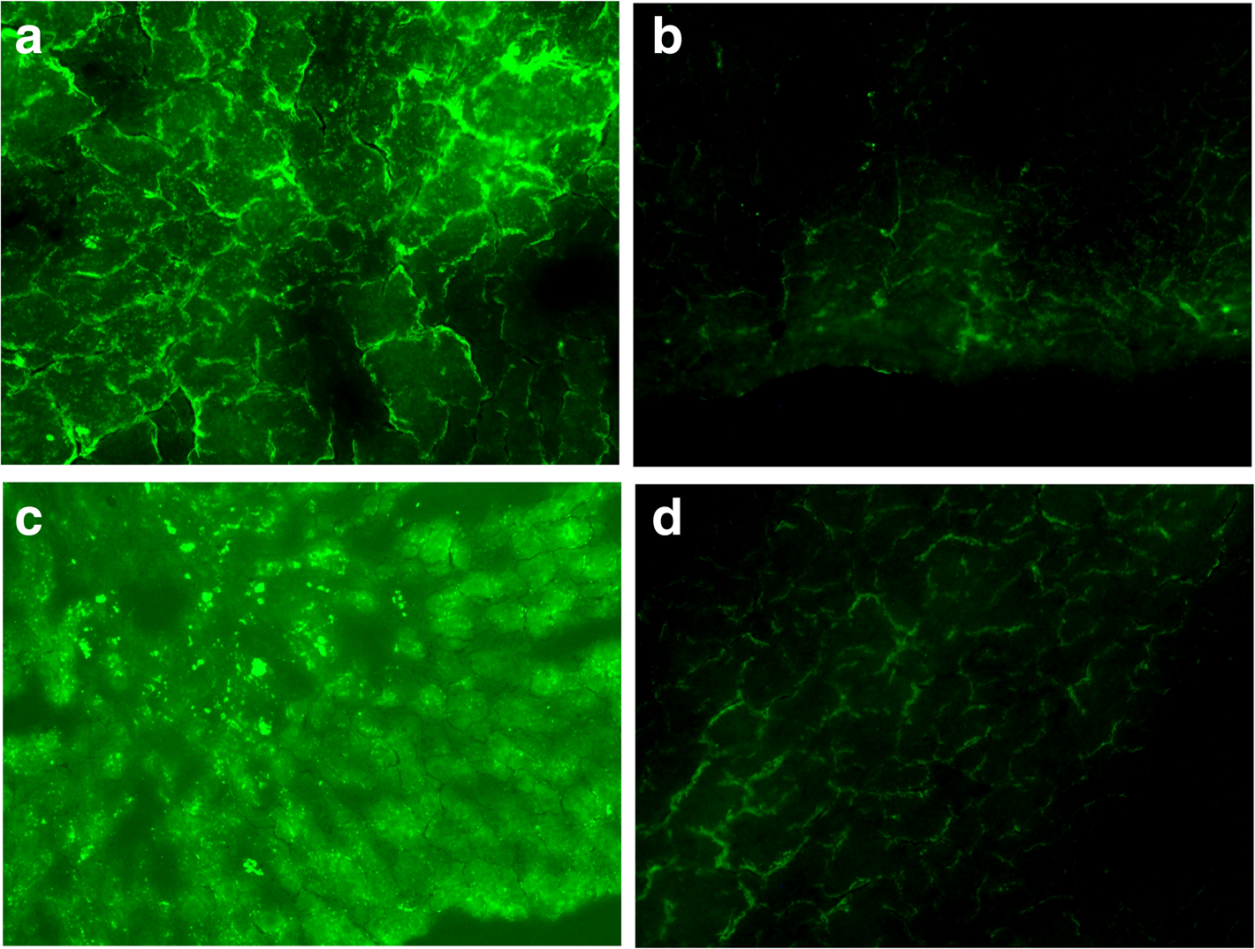
GFP fluorescence deposits in hepatic cells. **a** Control A group. **b** Control B group. **c** Experimental C group. **d** Experimental D group

GFP fluorescence deposits occurred in hepatic cells. One-way ANOVA revealed that the MOD values of GFP in the livers of different groups were not significantly different (*P* = 0.176) (Fig. 5, Table 2).

#### MOD of GFP in four organs of rabbits in each group

In the control A group and the experimental C group, no GFP fluorescence deposits occurred in the rabbit bladders. The MOD values of GFP in the other organs were not significantly different (*P* > 0.05).

The MOD values of GFP in the four organs were not significantly different in the control B group (*P* = 0.085).

In the experimental D group, one-way ANOVA and Tamhane’s T2 test (heterogeneity of variance, *P* < 0.05) revealed that the MOD values of GFP in the four organs in rabbits were significantly different (*P* < 0.05). The MOD values of GFP in the oviduct were higher than in the uterus and liver (*P* = 0.038, *P* = 0.012). The MOD values of GFP in the bladder were lower than in the liver (*P* = 0.024). The MOD values of GFP in the other organs were not significantly different (*P* > 0.05) (Table 3).

**Table 3:** Mean optical density of GFP in different organs

Organ	Control A group (*n* = 5)	Control B group (*n* = 5)	Experimental C group (*n* = 6)	Experimental D group (*n* = 6)
Oviduct	56.95 ± 2.26	62.45 ± 3.93	59.29 ± 7.58	82.94 ± 14.37
Uterus	40.03 ± 22.78	54.71 ± 0.85	37.22 ± 28.85	57.91 ± 9.53
Bladder	0.00 ± 0.00	49.34 ± 2.40	0.00 ± 0.00	51.86 ± 6.45
Liver	80.62 ± 13.49	66.87 ± 21.05	69.43 ± 3.50	64.59 ± 1.45
*F*	32.665	2.635	25.164	12.746
*P*	0.000	0.085	0.000	0.000

*GFP* green fluorescent protein

### Fluorescent colocalization of CK7 and WJMSCs in the oviduct

**Fig. 6:**
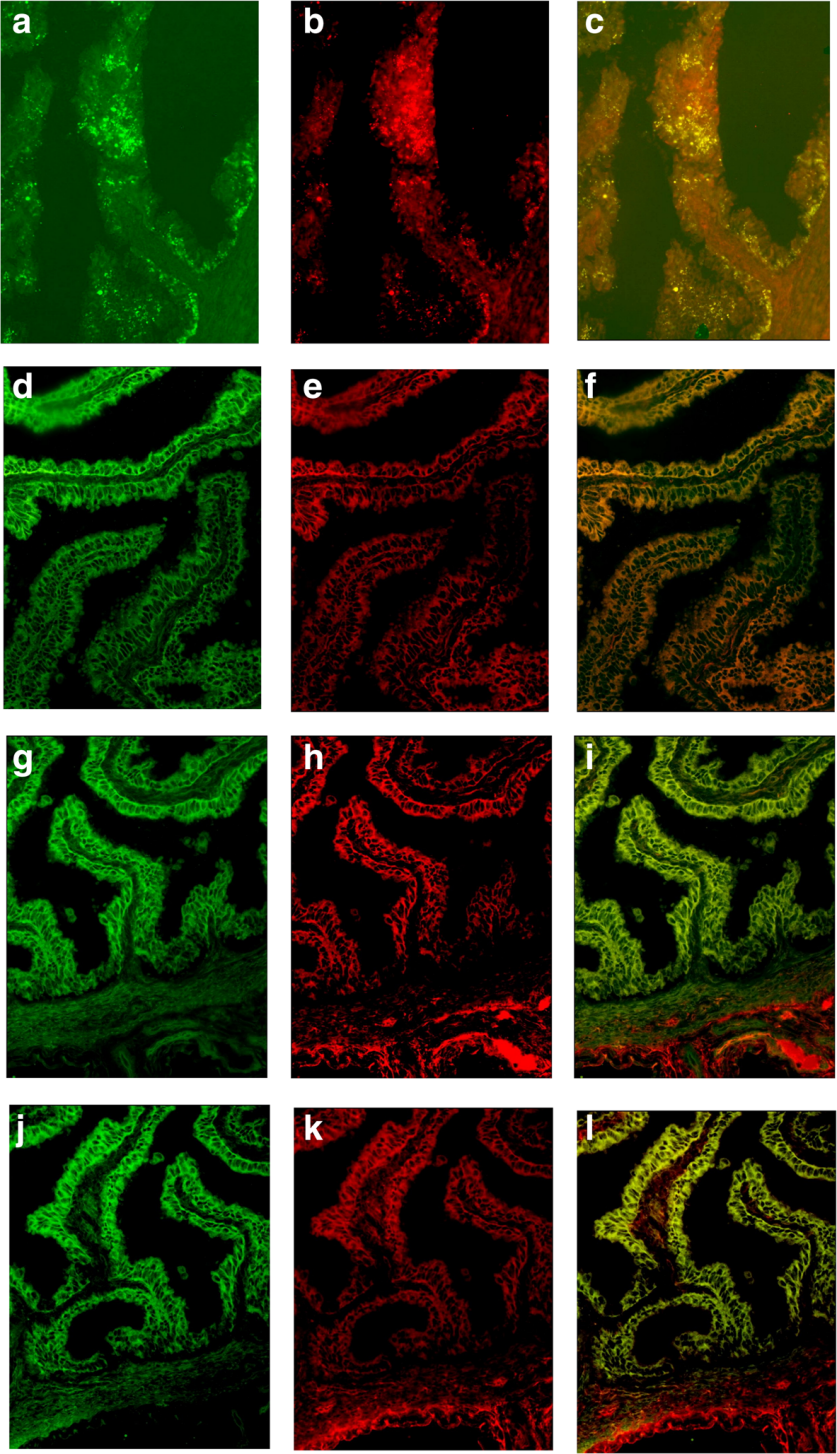
CK7-positive staining in the cytoplasm. Observed red fluorescence of CK7 overlapped with green fluorescence of WJMSCs with GFP in tubal epithelium. Yellow fluorescence of colocalization of CK7 and WJMSCs observed in all four groups. **a–c** Control A group. **d–f** Control B group. **g–i** Experimental C group. **j–l** Experimental D group

CK7 immunofluorescence staining was performed on oviduct tissues to evaluate whether WJMSCs promoted damaged epithelial cell growth. The results demonstrated CK7-positive cells in the cytoplasm of tubal epithelium. The red fluorescence of CK7 overlapped with the green fluorescence of WJMSCs in tubal epithelium. Yellow fluorescence indicative of the colocalization of CK7 and WJMSCs was observed in all four groups, especially in the experimental D group. One-way ANOVA and the LSD test (homogeneity of variance, *P* > 0.05) revealed that the MOD values of CK7 in tubal epithelium were significantly different (*P* < 0.05). The MOD values of CK7 in the experimental D group were higher than in the experimental C group and the control A group (*P* = 0.000, *P* = 0.000). The MOD values of CK7 in the control B group were higher than in the experimental C group and the control A group (*P* = 0.000, *P* = 0.000). The MOD values of CK7 in the other groups were not significantly different (*P* > 0.05) (Fig. 6, Table 4).

**Table 4:** Mean optical density of CK7 in rabbit tubal epithelium

Group	*n*	CK7 MOD	Compared with respective control group	Compared with experimental group
			P1	P2
Control A	5	49.73 ± 1.60		
Control B	5	61.31 ± 1.75		
Experimental C	6	50.50 ± 4.82	0.745	
Experimental D	6	66.22 ± 5.17	0.052	0.000

*F* = 24.683, *P* = 0.000

*CK7* cytokeratin 7, *MOD* mean optical density

## Discussion

Green fluorescent proteins and their variants and homologs of different colors are used in a variety of applications to investigate the organization and function of living systems [21]. A previous study used DiI, BrdU, and adenovirus-carrying GFP to label adipose-derived stem cells (ASCs), and the labeling efficiencies were compared at different time points and passages using fluorescence microscopy [22]. The results demonstrated that 100% and 90% of the ASCs emitted red fluorescence in the cytoplasm with no fluorescence in the nuclei 48 h after DiI and BrdU staining, respectively, but the fluorescence intensity declined quickly after cell passaging. Cell labeling with GFP adenovirus demonstrated a more stable labeling efficiency, and green fluorescence was detected 24 h after labeling. Greater than 90% of the ASCs remained positively stained without an obvious attenuation of fluorescence intensity 5 days later, even after cell passaging. Ma et al. [23] compared the efficiency of transfection of marrow stem cells using a lentiviral vector and an adenoviral vector. They found that the transfection efficiency of the BMP2 gene by lentivirus was significantly higher than that by adenovirus. It is worth noting that previous studies showed that GFP-carrying lentivirus had no adverse effect on the differentiation function and death rate of MSCs, laying the foundation for a valid method for the long term in vivo and in vitro [24–26]. Additionally, Tsai et al. [27] and Yu et al. [28] demonstrated that lentivirus-mediated transfection has no influence on the secretion function of MSCs. The transfection efficiency of WJMSCs transduced with LV3-GFP-PURO lentivirus was 96.7% in our study. The cell proliferation ability of WJMSCs in the transfer group and the untransfected group was not significantly different (*P* > 0.05), which is consistent with previous research [29]. These results demonstrated that lentivirus with the GFP gene was efficiently transfected and did not influence the proliferation ability of WJMSCs, within limits. Therefore, this method is applicable for labeling and tracking in vivo.

A recent study revealed that osteoblasts transfected with GFP using an adenovirus vector were expressed in vitro and were traceable in vivo. The authors found that the GFP was obviously expressed in nude mice at 4 and 8 weeks [30]. Another study injected neural stem cells with GFP into the paracelein of mice. The authors found that GFP fluorescence deposits occurred most significantly in the paracelein after 14 days. The GFP fluorescence decreased gradually and finally disappeared [31]. Rabbits in the control group were injected with a 1 × 10^6^/ml suspension of GFP-labeled WJMSCs once weekly for 3 weeks in our study. One week after the last perfusion, the oviduct, uterus, bladder, and liver were sampled for examination. We observed GFP in all four organs examined, especially in the oviduct in the experimental D group. However, we did not sample tissue after the last WJMSC perfusion, and we could not evaluate the long-term efficiency of GFP.

Gallatin et al. [32] first described lymphocyte homing to lymph nodes in vivo in 1983. MSC homing was defined subsequently as the arrest of MSCs within the vasculature of a tissue followed by transmigration across the endothelium. The exact mechanism of migration and homing characteristics of MSCs is still unknown. Worthy of note is that the influencing factors of homing characteristics, such as oxygen condition and some growth factors, were widely reported [33]. At present, some studies have shown that the mechanism may be decided by certain chemokines and their receptors [34]. Matrix metalloproteases (MMP) also play an important role in MSC migration [35]. Another important role is perfusion of MSCs. MSC therapy in clinics or experiments was often performed via intravascular injection with the hope that the cells would reach the lesion site through the circulation because of the homing function. However, previous studies of MSC homing reported that intravascular injections produced low MSC detection rates in target organs, which affected the curative effect of MSCs. Barbash et al. [36] found that systemic intravenous delivery of BM-MSCs to rats after myocardial infarction was limited by the entrapment of the donor cells in the lungs. Direct left ventricular cavity infusion enhanced the migration and colonization of the cells preferentially to the ischemic myocardium. In our study, the MOD values of GFP in the oviduct of the experimental D group were higher than those in the control A group and the experimental C group (*P* = 0.037, *P* = 0.047). The MOD values of GFP in the oviduct of the experimental D group were higher than those in the control A group, but this difference was not statistically significant. In the experimental D group, the MOD values of GFP in the oviduct of rabbits were higher than in the uterus and liver (*P* = 0.038, *P* = 0.012). These results suggest that the WJMSCs could home to the injured oviduct with limited efficiency. The efficiency was influenced by the perfusion method (e.g., artery, vein, or local perfusion), age, and passage number of the cells, and culture conditions [37]. Vaginal local perfusion provided direct contact of WJMSCs with injured tubal epithelium. The injured tubal epithelium may secrete chemotactic factors and adhesion molecules to attract WJMSCs to colonize and play a role in repair. This method may reduce the delay of WJMSCs that was observed in other organs or tissues. Our results demonstrated that future applications of WJMSCs should incorporate local or injury application as the preferred method to increase healing effectiveness.

A previous study directly injected isolated MSCs into adult mouse hearts, and MSCs were detected in the lung, small intestine, and stomach [38]. This study demonstrated that MSCs homed to injured sections and reached normal tissues. Our study injected WJMSCs into female rabbits in control and experimental groups via the ear vein and/or genital tract perfusion. The WJMSCs were found in the injured oviduct and the normal tissues (e.g., uterus, livers, bladder). No GFP fluorescence deposits occurred in the bladder of the control A group or experimental C group. The MOD values of GFP in the uterus in the control B group and the experimental D group were higher than in the control A group and the experimental C group, but this difference was not statistically significant (*P* = 0.217). These results demonstrated that the local injection of WJMSCs increased colonization in organs near the genital tract. However, the detailed mechanism requires further future research. The MOD values of GFP in the bladder were lower than in the liver in the control A group, the experimental C group, and the experimental D group (*P* = 0.000, *P* = 0.000, *P* = 0.024). This trend suggests that the liver, which is an organ with more blood flow than the bladder, may attract more WJMSCs. Additionally, although without significant difference, the MOD values of GFP in the livers of the control A group and the experimental C group, which included vein injections, were higher, especially in the control A group, than in the control B group and the experimental D group. This result suggests that future applications of WJMSCs via blood vessel perfusion would reduce the retention volume of WJMSCs in other normal organs, especially organs with a rich blood flow, which is very important.

Epithelial tissues of the oviduct play a very important role in reproductive processes. Cytokeratin is a special marker in the epithelial tissue of the female genital tract. It is a member of the cytoskeleton that provides crucial mechanical support [39, 40]. Many studies used cytokeratin to identify primary epithelial cells. The epithelial cells in the oviduct express CK7 [41]. It is known that WJMSCs express pan-cytokeratin but not CK7 [42]. Our research showed that colocalization of CK7 and WJMSCs was observed in tubal epithelium in all four groups, especially in the experimental D group. From the colocalization picture we could see that WJMSCs/CK7 appeared alone or overlapped with the other. This result suggested that parts of new epithelial cells in the oviducts were differentiated from parts of transplanted WJMSCs in our study. Additionally, the MOD values of CK7 in tubal epithelium were significantly different in the four groups (*P* < 0.05). The MOD values of CK7 in the experimental D group were higher than those in the experimental C group (*P* = 0.000). The MOD values of CK7 in the control B group were higher than in the control A group (*P* = 0.000). These results demonstrated that vaginal local transplantation of WJMSCs into oviduct epithelium was easier to promote epithelial cell growth. The mechanism of inflammation therapy of WJMSCs may be divided into two parts. First, WJMSCs differentiated into epithelial cells directly. This mechanism was demonstrated in previous studies [43, 44]. Second, WJMSCs also promoted epithelial cell growth via a secretion function, such as increased secretion of IL-10, and the inhibitory effect of proinflammatory factors improved the microenvironment of injured sections [45, 46].

### Limitation

Although the main purpose of our study was to trace WJMSCs in vivo via using a chronic salpingitis model, we have not confirmed the survival time and secretion function of WJMSCs after engraftment. In our further study, this field will be the main problem to be solved. Additionally, an antibiotic medication group could be better to compare the therapeutic effect of WJMSCs. Furthermore, GFP sliders without DAPI are a main drawback of our study. Another major limitation of this study was that the sample size was ultimately not large enough to obtain a significant difference. Therefore, further investigation is currently underway with a larger sample size.

## Conclusion

Lentivirus with a GFP gene was an efficient transfection agent that did not influence the proliferation ability of WJMSCs within limits. This method is applicable for labeling and tracking in vivo. Notably, WJMSCs exhibited homing characteristics and migrated to the injured oviduct to promote epithelial cell growth. The transplanted WJMSCs could differentiate into epithelial cells. Local treatment provided higher efficiency.

## Abbreviations

ASC: Adipose-derived stem cell
GFP: Green fluorescent protein
HCG: Human chorionic gonadotropin
MHC: Main histocompatibility complex
MMP: Matrix metalloproteases
MOD: Mean optical density
MSC: Mesenchymal stem cell
WJMSC: Wharton’s jelly-derived mesenchymal stem cell
